# Intergenerational Homesharing: Community Cohered by Story

**DOI:** 10.1093/geroni/igaa057.1734

**Published:** 2020-12-16

**Authors:** Rachel Wenrick, Kirsten Kaschock

**Affiliations:** Drexel University, Philadelphia, Pennsylvania, United States

## Abstract

Drexel’s Writers Room is a university-community literary arts program engaged in creative placemaking and art for social justice. Since 2014, our work has focused on two major themes: how we, as a community of writers ages 18-80, can create and connect through story; and how together we can address some of the changes we are seeing in the local neighborhood around Drexel’s campus. This presentation will provide an overview of how the method of co-creation, core to our program since its inception, has allowed us to develop the concept for a writers house and intergenerational homesharing network as an aging-in-place and anti-displacement strategy; build the extensive cross-sector partner network needed to implement it, and earn crucial neighborhood and institutional support. The goal is for attendees to leave with ideas for how this concept might be adapted in their own communities.

